# Open vs. Robot-Assisted Artificial Urinary Sphincter Implantation in Women with Stress Urinary Incontinence: A Multicenter Comparative Study

**DOI:** 10.3390/jcm14010284

**Published:** 2025-01-06

**Authors:** Alexandre Dubois, Grégoire Capon, Olivier Belas, Adrien Vidart, Andrea Manunta, Juliette Hascoet, Lucas Freton, Frederic Thibault, Vincent Cardot, Frédéric Dubois, Luc Corbel, Emmanuel Della Negra, François Haab, Laurence Peyrat, Jean-Nicolas Cornu, Philippe Grise, Aurélien Descazeaud, Georges Fournier, Benoit Peyronnet

**Affiliations:** 1 Department of Urology, University of Rennes, 35000 Rennes, France; 2 Department of Urology, University of Bordeaux, 33404 Bordeaux, France; 3 Department of Urology, Pole Le Mans Sud, 72100 Le Mans, France; 4 Department of Urology, Foch Hospital, 92150 Suresenes, France; 5 Department of Urology, Clinique Mutualiste La Sagesse, 35000 Rennes, France; 6 Department of Urology, Hopital Robert Schuman, 57070 Vantoux, France; 7 Department of Urology, Clinique Bizet, 75116 Paris, France; 8 Department of Urology, Hospital Privé Saint-Grégoire, 35760 Saint-Grégoire, France; 9 Department of Urology, Centre Briochin d’Urologie de l’Hopital Privé Cotes-D’armor, 22190 Plerin, France; 10 Department of Urology, Institut Montsouris, 75014 Paris, France; 11 Department of Urology, Hopital Diaconesses Croix-Saint Simon, 75020 Paris, France; 12 Department of Urology, University of Rouen, 76031 Rouen, France; 13 Department of Urology, University of Limoges, 87000 Limoges, France; 14 Department of Urology, University of Brest, 29609 Brest, France

**Keywords:** artificial urinary sphincter, female, stress urinary incontinence, intrinsic sphincter deficiency, robotic, robot-assisted, open, comparison

## Abstract

**Background:** The artificial urinary sphincter has been an effective treatment for stress urinary incontinence caused by intrinsic sphincter deficiency in women. However, the use of this device has been limited by the technical difficulties and risks associated with the open implantation procedure. Preliminary studies using robotic techniques have shown promising results, but only one small study has compared robotic to open procedures. This study aims to compare the outcomes of robotic and open artificial urinary sphincter implantation in women with stress urinary incontinence due to intrinsic sphincter deficiency in a large multicenter cohort. **Methods**: Data were collected retrospectively from female patients who underwent open or robot-assisted artificial urinary sphincter implantation from 2006 to 2020 at 12 urology departments. The primary outcome was the rate of complications within 30 days after surgery, graded using the Clavien-Dindo Classification. Perioperative and functional outcomes were compared between the two groups. **Results:** A total of 135 patients were included, with 71 in the robotic group and 64 in the open group. The open group had a higher rate of intraoperative complications (27.4% vs. 12.7%; *p* = 0.03) and postoperative complications (46.8% vs. 15.5%; *p* < 0.0001). More patients in the robotic group achieved full continence (83.3% vs. 62.3%; *p* = 0.01). The open group had higher explantation (27.4% vs. 1.4%; *p* < 0.0001) and revision rates (17.5% vs. 5.6%; *p* = 0.02). The estimated 1-year explantation-free survival rate was higher in the robotic group. (98.6% vs. 78.3%; *p* = 0.001). **Conclusions:** Robot-assisted implantation may reduce perioperative morbidity and improve functional outcomes compared to open implantation in women with stress urinary incontinence.

## 1. Introduction

Female stress urinary incontinence (SUI) is a highly prevalent disorder severely altering the quality of life of affected individuals [1,2,3]. Female outlet resistance relies mostly on two mechanisms: the internal and external urinary sphincters (intrinsic mechanism) and the urethral hammock and other anatomical support of the urethra (external mechanism) [4,5]. Inherently, female SUI is underpinned by two main pathophysiological contributors: urethral hypermobility (failure of the extrinsic mechanism) and intrinsic sphincter deficiency (ISD; failure of the intrinsic mechanism). The latter is mostly encountered in patients who failed previous anti-incontinence procedures, but also in specific populations such as in elderly, irradiated or neurogenic women [6,7]. The artificial urinary sphincter (AUS) has been reported as an effective treatment option in female patients with SUI due to ISD, but its use has long been hindered by the technical challenge and morbidity of the open implantation technique [8,9].

Several initial studies have highlighted the use of a robotic technique for AUS implantation in female patients with SUI caused by ISD, yielding promising outcomes [10,11]. However, to date, only one small-sample single-center study has sought to compare the outcomes of robotic AUS implantation with those of open AUS implantation in female patients [12]. The aim of the present study was to compare the outcomes of robotic vs. open AUS implantation in women with ISD-predominant SUI in a large multicenter cohort.

## 2. Methods

### 2.1. Study Design

The retrospective study included data from all female patients who underwent open or robot-assisted AUS implantation for SUI caused by ISD between 2006 and 2020 at 12 urology departments. AUS was presented as an option to all female patients with SUI due to ISD identified by a positive cough stress test with a fixed or poorly mobile urethra on physical examination. Urodynamic investigation was performed in all of these patients, and a low maximum urethral closure pressure (MUP) was deemed as a co-argument of ISD [13]. The alternative offered to these patients were a second synthetic midurethral sling (MUS), periurethral adjustable continence therapy (ACT) silicone balloons or bulking agents as per the national guidelines [14]. Some departments were using an exclusively open approach for AUS implantation, and some moved from an open to a robotic approach in 2013, 2014 or 2015. Once they switched to the robotic approach, no further open implantations were performed.

At the start of the study period, all surgeons performing robotic implantation had little to no (<20 cases) experience of AUS implantation. In contrast, in the open group, two of the 5 surgeons involved had performed over 20 AUS implantations. All implanted artificial urinary sphincter were AMS800 devices by Boston Scientific ^®^, Marlborough, MA, USA.

This study was approved by the CNIL (Comité National Informatique et Liberté, CNIL 2235498v0), and was conducted following the principles of the Helsinki declaration.

The following baseline characteristics were documented in a dedicated computerized dataset for all patients: age at the time of AUS implantation, ASA score, body mass index (BMI), etiology of incontinence (neurogenic vs. non-neurogenic), history of radiotherapy, history of previous anti-continence surgery, number of pads used per day, type of pad, presence of preoperative urgency, maximum free urinary flow rate (Qmax), post-void residual (PVR). Preoperative urodynamic parameters, including detrusor overactivity and maximum urethral closure pressure, were also collected.

### 2.2. Perioperative Management

All patients had urinalysis prior to surgery. If the preoperative urine culture was positive (≥10^3^ CFU/mL), the patient received antibiotics adjusted to the antibiogram starting 48 h prior to surgery. All patients received perioperative antibioprophylaxis (2 g of cephalosporin or 2 g of amoxicillin-clavulanic acid). The device was deactivated at the end of the procedure.

The urethral catheter was removed in the operative room or on day 1 postoperatively, except in case of bladder injury, where it was kept for 10 to 14 days.

The AUS was activated at six weeks postoperatively.

### 2.3. Surgical Techniques

#### 2.3.1. Robot-Assisted Laparoscopic Implantation

All robot-assisted AUS implantation used an anterior transperitoneal approach using the Intuitive Da Vinci Si, X or Xi surgical robot according to the technique previously described [15]. Briefly, the four-arm Da Vinci robot was placed in a right-side docking position. The patient was placed in a 23° Trendelenburg position with access to the vagina. A 14 French urethral catheter was inserted, and five ports were placed at the level of the umbilicus in a straight line according to the usual set-up for robotic pelvic procedures.

The peritoneum was opened, and the bladder was dropped down from the anterior abdominal wall to enter the Retzius space. After reaching the endopelvic fascia on both sides and removing or dividing all the material from previous anti-incontinence procedures (e.g., midurethral slings, Burch colposuspension stitches, pubovaginal sling), the posterior aspect of the bladder neck was dissected. The assistant’s surgeon placed a finger in each of the anterior vaginal fornixes next to the bladder neck allowing the robotic surgeon to progressively dissect the vesicovaginal plane bluntly.

After dissecting the bladder neck circumferentially, the measuring tape was inserted to size the bladder neck and choose the appropriate cuff size. After placing the cuff around the bladder neck, the pressure-regulating balloon was placed through a 3 cm suprapubic incision. The cuff’s tubing was extracted through the same suprapubic incision. Finally, a space was created in the labia majora from the suprapubic incision to insert the pump, and the tubing’s connections were carried out.

#### 2.3.2. Open Implantation

For open AUS implantation, we used the technique previously described by most authors [9]. After placing a 16 French Foley urethral catheter, we used an abdominal approach through a Pfannenstiel incision. The Retzius space was then dissected until the bladder neck, and the endopelvic fascia was opened on both sides of the urethra as described by Costa et al. [16]. Below the urethral catheter’s balloon, which was used as a landmark, the bladder was dissected from the vagina below the periurethral fascia. The surgeon introduced two fingers of his left hand into the vagina to help the dissection that he kept performing with his right hand.

A leak test with saline and methylene blue was performed to rule out any bladder injury. Contrary to the previously described technique, the bladder dome was never opened. The measurement of the bladder neck circumference and the device implantation were performed in a similar manner to the robotic approach described above.

### 2.4. Outcomes of Interest

The primary endpoint was the proportion of 30-day postoperative complications graded according to the Clavien-Dindo Classification [17].

The secondary outcomes of interest were as follows: (I) complete continence defined as wearing no pad at all; (II) the explantation and revision rates; and (III) the device explantation-free survival rate.

The following perioperative outcomes were also collected: mean operative time, estimated blood loss, length of hospital stay, intraoperative complications (vaginal or bladder neck injury). The perioperative and functional outcomes between the open and robotic groups were compared.

### 2.5. Statistical Analysis

Means and standard deviations were reported for continuous variables, medians and ranges for categorical variables and proportions for nominal variables. Comparisons between groups were performed using the χ^2^ test or Fisher’s exact test for discrete variables and the Mann–Whitney test for continuous variables as appropriate.

Probability of revision-free and explantation-free survivals was estimated using the Kaplan–Meier method. The last follow-up was defined by the most recent date on which information were collected, either during an outpatient clinic visit or a phone call to update the patient’s data. Patients without any event (revision or explantation) during the study period were censored at the date of the last follow-up.

Statistical analyses were performed using JMP v.12.0 software (SAS Institute Inc., Cary, NC, USA). All tests were two-sided with *p* < 0.05 as a threshold to define statistical significance.

## 3. Results

### 3.1. Patients’ Characteristics

Over the study period, 135 patients were included: 71 in the robotic group and 64 in the open group. Most of the patients’ baseline characteristics did not differ significantly (see Table 1). The median age was 66.5 years in the open group vs. 68 years in the robotic group (*p* = 0.57). There were comparable proportions of neurogenic SUI patients in both groups (10.9% vs. 6%; *p* = 0.32), but there were significantly more radiated patients in the open group (6.2% vs. 0%; *p* = 0.03). The vast majority of patients in both groups had undergone at least one previous anti-incontinence procedure (89% vs. 90.1%; *p* = 0.95).

The distribution of robotic AUS implantations across centers was as follows: Center 1: 23 patients, Center 2: 14 patients, Center 3: 12 patients, Center 4: 9 patients, and Centers 5 and 6: 4 patients each. Additionally, Center 7 had 3 patients, and Center 8 had 2 patients. For open AUS implantations, the distribution was as follows: Center 1: 16 patients, Center 2: 15 patients, Center 3: 14 patients, Center 4: 10 patients, and Center 5: 9 patients.

### 3.2. Perioperative Outcomes

The mean operative time was longer in the robotic group (179.9 vs. 126.2 min; *p* < 0.0001). The rate of intraoperative complications was higher (i.e., intraoperative bladder neck and/or vaginal injury) as were the rates in the open group (12.7% vs. 27.4%; *p* = 0.03) and the rate of postoperative complications (15.5% vs. 46.8%; *p* < 0.0001) and major Clavien Grade ≥ 3 complications (2.8% vs. 17.2%; *p* = 0.01). There was no Clavien Grade 4 or 5 complication in either of the groups. The estimated blood loss was more important in the open AUS group (16.2 vs. 164.1 mL; *p* < 0.0001). The mean length of hospital stay was 6.5 days in the open group vs. 4.1 days in the robotic group (*p* = 0.002) (See Table 2).

### 3.3. Functional Outcomes and Device Survival

**Figure 1:**
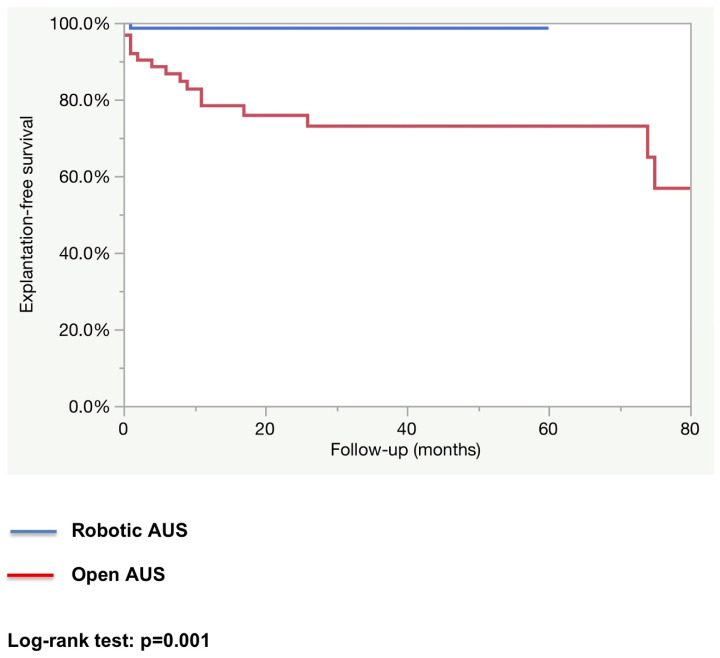
Device explantation-free survival.

**Figure 2:**
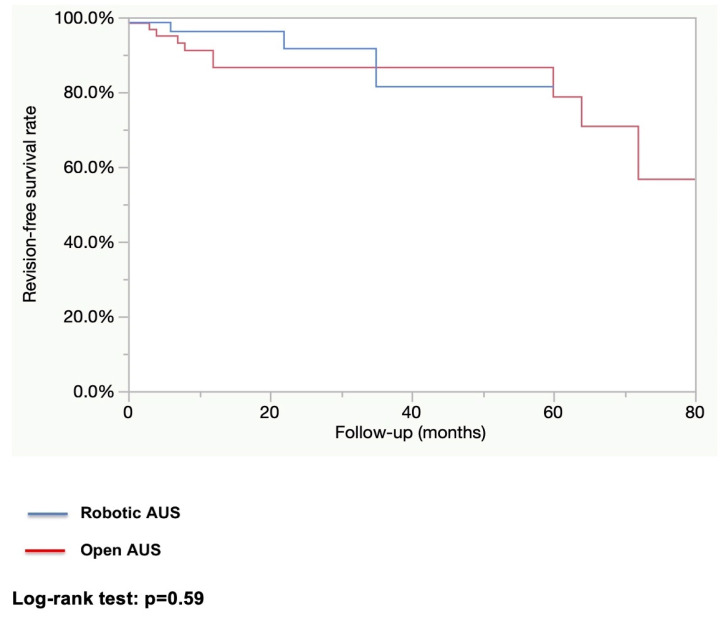
Device revision-free survival.

After a median follow-up of 12.2 months in the robotic group and 25.5 months in the open group, the rate of patients fully continent (i.e., 0 pad per day) was higher in the robotic group (83.3% vs. 62.3%; *p* = 0.01). The explantation rate as well as the revision rate were higher in the open AUS group (explantation rate: 27.4% vs. 1.4%; *p* < 0.0001, and revision rate: 17.5% vs. 5.6%; *p* = 0.02). (See Table 3). The estimated 1-year explantation-free survival rate was higher in the robotic group (98.6% vs. 78.3%; *p* = 0.001; Figure 1). The estimated 1-year revision-free survival was similar in both groups (86.6% vs. 96.2%; *p* = 0.59; Figure 2). All explantations were due to device infection or erosion. Specific causes for revision were not collected.

## 4. Discussion

Since the first implantation of an AUS in a female patient in 1973, excellent results in terms of continence have been described. However, the use of the device has failed to spread over the years in the female population. This can be explained on one hand by the high reported rates of complication inherent to the technical challenge of female AUS implantation using an open approach. On the other hand, it can also be explained by the psychological impact of the device for patients and the anatomical constraints such as the position of the pump and its manipulation.

The advent of robotic surgery since the early 2000s has revolutionized many surgical procedures [18]. The Da Vinci robotic system by Intuitive is the most commonly used surgical robot. Its enhanced 3D vision in the pelvis and its endowrist technology makes it particularly useful to intervene in narrow and deep cavities, which is particularly convenient for pelvic surgical procedures such as female AUS implantation [19]. The present study provides a comparison between open and robot-assisted AUS. We found fewer peri- and postoperative complications, a lower explantation rate and less estimated blood loss in the robotic implantation group. The complete continence rate was also higher in the robot-assisted implantation group. We observed a longer mean operative time in the robotic group, likely attributable to the innovative nature of the technique, which required adjustments, and the early stages of the learning curve at some centers.

These results can be explained by the advantages of the surgical robot. Firstly, contrary to the open technique where dissection was mostly performed blindly by palpation and digital feeling with one or two fingers in the vagina, the robotic approach offers a 3D-enhanced vision into the pelvis. This allows for conducting the dissection of the posterior aspect of the bladder neck under constant vision conversely to what was performed in the open technique, therefore minimizing the risk of intraoperative bladder neck or vaginal injury but also allowing early identification of these injuries when they occur. Hence, rather than worsening missed injuries as in open AUS implantation, these injuries can be repaired carefully under direct vision.

Contrary to standard laparoscopic instruments, the endowrist technology, by providing a seven-degree movement like a human wrist does in open surgery, is a key asset over open and laparoscopic techniques [20]. Considering the small size of the instruments, it offers a great range of motion and allows for fully wristed dexterity in highly constrained spaces, which is important for bladder neck dissection as long open instruments cannot be flexed in the bony pelvis due to the close contact between the bladder neck and the pubic bone [21]. The use of pneumoperitoneum while dissecting under constant vision and with the help of coagulation instruments could explain the lower estimated blood loss observed in the robotic group.

The better perioperative and postoperative outcomes found in the robot-assisted implantation group, despite the overall lower level of experience of the surgeons in this group, emphasize the educational power of the robotic approach as well as the better reproducibility of the surgery. All of the following explanations regarding the advantages of the robotic approach are based on our experience and represent the recommendations we would offer to surgeons planning to perform robotic AUS implantation. Thanks to videoscopy, the main surgical steps can be visualized intraoperatively and also remotely, at any time, on surgical video platforms, and the required fundamental skills can be learned before surgery on a dedicated simulator. Moreover, Intuitive introduced the dual console interface in 2009, which enables integrated teaching and direct supervision while maintaining the patient’s safety without drastically increasing the operative time [22,23]. Remote supervision through telementoring may become an additional tool to further improve the safe teaching of challenging surgical procedures such as robotic female AUS implantation.

It is harder to explain the better functional outcomes found in the robotic group. However, we might speculate that a more standardized and reproductible technique could lead to better cuff placement. With constant vision, surgeons might dissect more accurately at the bladder neck, which has thicker tissues that may reduce the risks of atrophy and erosion over time. Beyond decreasing perioperative complications and hence the explantation rate, this ideal placement may also ensure that the cuff occupies an optimum position for better function.

Another explanation lies in the fact that some centers first performed implantations using the open approach and then the robotic approach. Once they switched to the robotic approach, no further open implantations were performed. The overall center’s experience with female AUS implantation was then higher during robot-assisted implantations.

Another explanation could be the absence of patients with a history of pelvic radiation therapy in the robot-assisted implantation group, as this has long been considered a major risk factor for AUS failure. We believe the low number of patients with a history of pelvic radiation therapy is due to the high risk of complications such as erosion, pubic bone infection, and fistula formation associated with the cuff placement around the bladder neck.

From a general standpoint, the robotic approach to AUS implantation in women has become more popular over the years, but the cost of this technology is still a limiting factor. The development of surgical robots by other companies is beginning in the field and may contribute to reducing the price and the cost of single-use instruments [24]. It could also help in developing new tools and aspects of this approach. As an example, the adoption of the single-port Intuitive surgical robot is expanding. This new feature brings the possibility of regionalizing surgeries focusing on the relevant anatomy, as it has been described in radical prostatectomy [25,26]. In the field of AUS implantation, it could help in the development of a reproducible preperitoneal approach that may enhance perioperative and postoperative outcomes.

The present study has several limitations that should be considered when analyzing our findings. To begin with, the retrospective nature of the study brings many potential biases. The multicentric nature of the study brings heterogeneity: some centers only implanted via the open approach, while others switched more or less rapidly to the robotic one. Moreover, only the anterior robotic approach implantation was evaluated in this study, while several teams have described a posterior robotic approach technique which was not evaluated in the present report [27,28]. The causes of revision were not recorded in our dataset and could therefore not be presented, which may be regarded as a limitation. Also, our analysis of the short to medium term may underestimate the overall number of erosions and/or revisions in both groups. Regarding functional outcome, our evaluation is limited because we only evaluated complete continence subjectively, i.e., as self-reported by the patient. The multicentric and retrospective nature of the study did not allow us to collect robust data on patients’ reported outcomes using validated questionnaires. Finally, the main limitation might be the shorter median follow-up time for the robotic implantation population. This shorter duration may result in an underestimation of explantation and revision in the long run. Therefore, further studies with larger populations and extended follow-up periods would be valuable to confirm our findings and provide a clearer understanding of erosion and revision rates over time, especially for the robot-assisted implantation technique.

## 5. Conclusions

The robot-assisted approach, even when performed by less experienced surgeons during their learning curve, may reduce perioperative morbidity associated with AUS implantation in women compared to the open approach and could potentially improve functional outcomes, likely due to a lower explantation rate. If confirmed by further studies, these findings may entail a larger adoption of the robotic approach for female AUS implantation and shed a new light on the role of the AUS in female SUI patients.

## Figures and Tables

**Table 1:** Population’s characteristics.

	Open AUS *n* = 64	Robotic AUS *n* = 71	*p*-Value
Median age (years)	66.5 (IQR: 59–74)	68 (IQR: 61–74)	0.57
Mean Body Mass Index (kg/m^2^)	28.5 (±4.9)	27.2 (±4.8)	0.13
ASA score			0.21
1	8 (12.9%)	14 (21.2%)	
2	36 (58.1%)	40 (60.6%)	
3	18 (29%)	12 (18.2%)	
Neurogenic SUI	7 (10.9%)	4 (6%)	0.32
History of radiation therapy	4 (6.2%)	0 (0%)	0.03
History of previous anti-incontinence procedure	57 (89%)	64 (90.1%)	0.95
History of previous synthetic midurethral sling	47 (74.6%)	57 (80.3%)	0.44
Mean Maximum urethral closure pressure (cmH₂O)	25.9 (±14.5)	22.8 (±8.2)	0.29
Detrusor overactivity on preoperative urodynamics	9 (15.5%)	6 (8.4%)	0.17

**Table 2:** Perioperative outcomes.

	Open AUS *n* = 64	Robotic AUS *n* = 71	*p*-Value
Mean operative time (min)	126.2 (±51.8)	179.9 (±48.5)	<0.0001
Intraoperative complications (Vaginal or bladder neck injury)	17 (27.4%)	9 (12.7%)	0.03
Mean estimated blood loss (mL)	164.1 (±194.1)	16.2 (±37.3)	<0.0001
30-day postoperative complications	29 (46.8%)	11 (15.5%)	<0.0001
Major postoperative complications (Clavien ≥ 3)	11 (17.2%)	2 (2.8%)	0.01
Mean length of hospital stay (days)	6.5 (±5.6)	4.1 (±1.9)	0.002

**Table 3:** Functional and device outcomes.

	Open AUS *n* = 64	Robotic AUS *n* = 71	*p*-Value
Complete continence (no pad)	40 (62.3%)	59 (83.3%)	0.01
Explantation	22 (27.4%)	1 (1.4%)	<0.0001
Revision	11 (17.5%)	4 (5.6%)	0.02
Median follow-up (months)	25.5 (IQR: 8–55)	12.2 (IQR: 4–22)	<0.0001

## Data Availability

The data that support the findings of this study are available on request from the corresponding author. The data are not publicly available due to privacy or ethical restrictions.
